# A new risk model based on a 11-m^6^A-related lncRNA signature for predicting prognosis and monitoring immunotherapy for gastric cancer

**DOI:** 10.1186/s12885-021-09062-2

**Published:** 2022-04-05

**Authors:** Liangliang Lei, Nannan Li, Pengfei Yuan, Dechun Liu

**Affiliations:** 1 Department of Gastrointestinal Surgery, The First Affiliated Hospital, and College of Clinical Medicine of Henan University of Science and Technology, No.24 Jinghua Road, Jianxi District, Luoyang, 471003 China; 2 Department of Ultrasonography, The Sixth People’s Hospital of Luoyang, Luoyang, 471003 China

**Keywords:** N^6^-methyladenosine, Long non-coding RNA, Gastric cancer, Prognosis, Immunotherapy

## Abstract

**Objective:**

N^6^-methyladenosine (m^6^A) mRNA modification triggers malignant behaviors of tumor cells and thereby drives malignant progression in gastric cancer (GC). However, data regarding the prognostic values of m^6^A RNA methylation-related long non-coding RNAs (lncRNAs) in GC are very limited in the literature. We aimed to investigate the prognostic potential of m^6^A-related lncRNAs in predicting prognosis and monitoring immunotherapy efficacy in GC patients.

**Methods:**

Transcriptome and clinical data were obtained from GC biopsies from Cancer Genome Atlas (TCGA). M^6^A-related lncRNAs associated with GC were identified by constructing a co-expression network, and the gene pairs differentially expressed in GC were selected using univariate analysis. We constructed a risk model based on prognosis-related lncRNA pairs selected using the LASSO algorithm and quantified the best cutoff by comparing the area under the curve (AUC) for risk stratification. A risk model with the optimal discrimination between high- and low-risk GC patients was established. Its feasibility for overall survival prediction and discrimination of clinicopathological features, tumor-infiltrating immune cells, and biomarkers of immune checkpoint inhibitors between high- and low-risk groups were assessed.

**Results:**

Finally, we identified 11 m^6^A-related lncRNA pairs associated with GC prognosis based on transcriptome analysis of 375 GC specimens and 32 normal tissues. A risk model was constructed with an AUC of 0.8790. We stratified GC patients into high- and low-risk groups at a cutoff of 1.442. As expected, patients in the low-risk group had longer overall survival versus the high-risk group. Infiltration of cancer-associated fibroblasts, endothelial cells, macrophages, particularly M2 macrophages, and monocytes was more severe in high-risk patients than low-risk individuals, who exhibited high CD4^+^ Th1 cell infiltration in GC. Altered expressions of immune-related genes were observed in both groups. PD-1 and LAG3 expressions were found higher in low-risk patients than high-risk patients. Immunotherapy, either single or combined use of PD-1 or CTLA4 inhibitors, had better efficacy in low-risk patients than high-risk patients.

**Conclusion:**

The new risk model based on a 11-m^6^A-related lncRNA signature can serve as an independent predictor for GC prognosis prediction and may aid in the development of personalized immunotherapy strategies for patients.

**Supplementary Information:**

The online version contains supplementary material available at 10.1186/s12885-021-09062-2.

## Introduction

GC is the fifth most common malignancy worldwide, with a high incidence of new cases of 1 million each year, according to the 2018 statistics [1]. In China, GC is the second-largest malignancy regarding both morbidity and mortality, just following lung cancer [2]. Despite advances in diagnostics and treatment, a substantial number of patients with new lesions failing to be detected are often confirmed until a late stage, facing higher risks of metastasis and failure of anti-cancer therapy. For all the efforts that have been made for a better prognosis, the average 5-year survival rate of GC is still around 30%, while that of early-to-middle stage patients surprisingly reaches 60% [3, 4]. Even the promising immune checkpoint inhibitors (ICIs), which are expected to offer greater clinical benefits to patients with various cancers, including GC, reveal limited efficacy in advanced patients. Identification of sensitive biomarkers for early diagnosis and prognosis prediction is urgently needed.

Immune checkpoints may impede T cell activation by triggering several inhibitory signaling pathways, which is a key mechanism responsible for tumor cell escape from host immune recognition and T cell-mediated killing. ICIs, which block immunosuppressive signals and boost the activity of immune cells, seem to become a “game changer” for cancer treatment in the twenty-first century. PD-1/PD-L1 inhibitors are the most effective drugs to boost clinical response to immunotherapy [5, 6]. However, the global, multicenter, phase 2 KEYNOTE-059 study ascertained a fair response of advanced GC to pembrolizumab, which was only approved for use as a third-line treatment [7]. In the phase 3 ATTRACTION-2 study, nivolumab significantly increased the survival rate of progressive GC patients after ineffective chemotherapy [8]. The 2021 Chinese Society of Clinical Oncology (CSCO) included immunotherapy for the first-line/second-line treatment of GC [9]. These shreds of evidence support the efficacy of ICIs in GC treatment, but more new drugs are needed to offer more options for advanced patients.

N^6^-methyladenosine (m^6^A) refers to methylation in the N^6^ position of adenosine. It frequently occurs in the RRACH sequence near the 3′ non-coding region, stop codon, and long internal exon [10]. This modification affects RNA fate in mammalian cells by dynamically and reversibly regulating the charge of RNA base, the secondary structure of RNA, and protein-RNA interaction to alter RNA transport, localization, translation, and degradation [11, 12]. Therefore, m^6^A-associated cancer development in various cancer types, including GC, has been intensively studied [13, 14]. Current GC studies primarily focus on m^6^A modification in protein-coding genes promoting prognosis [15]. But the effect of m^6^A-associated non-coding RNAs on GC and their prognostic value, yet little explored, is important for immune monitoring and long-term remission in these patients. LncRNAs represent a type of transcript exceeding 200 nucleotides of length, accounting for about 90% of the human transcriptome [16]. They generally do not encode proteins yet regulate RNA expression at the epigenetic, transcriptional, or post-transcriptional level [17]. M^6^A lncRNA modification is associated with a shift towards an immune-inflamed phenotype in cancers, characterized by immune cell infiltration into the tumor microenvironment (TME) [18, 19], suggesting that it may effectively determine tumor cell survival and efficacy of anti-cancer therapies. M^6^A-related lncRNAs signatures have been shown to have pronounced correlations with the TME and expressions of critical immune checkpoints in hepatocellular carcinoma [20] and lung adenocarcinoma [21]. However, these models for prognosis prediction or assessment of immune cell infiltration and expressions of immune checkpoints in GC have not been reported elsewhere.

In this study, we aimed to develop a new risk model for various predictions for GC patients and constructed a new iteration algorithm for optimal gene-pairing strategies. Predictive performances of the risk model in low- and high-risk patients stratified by the optimal cutoff calculated based on AUCs were assessed. For the feasibility of this model, we evaluated whether it could discriminate differences in immune cell subpopulations between low- and high-risk groups. Correlation analyses were performed to determine whether there were relationships between the new risk model we constructed and known biomarkers for predicting the efficacy of immunosuppressive therapy. Overall, this study will provide fresh insights into the roles of m^6^A-related lncRNAs in the prediction of GC prognosis and immunotherapy efficacy.

## Methods

### Transcriptome analysis for m^6^A-related lncRNAs in GC

We integrated clinical and transcriptome (or RNA-seq) data from TCGA (https://gdc.cancer.gov/) in March 2021 for identifying m^6^A-related lncRNAs differentially expressed between GC and healthy controls. Patients were included if they fulfilled the following criteria: (1) they were histopathologically diagnosed; (2) tissue samples were obtained by surgery or tissue biopsy prior to other clinical treatments; and (3) clinical information as tissue ID, age, sex, grade, stage, and follow-up time was complete. Patients younger than 18 years or with incomplete clinical information were excluded, and patients not participating in any follow-up visits were also ruled out. Ensembl IDs from the pooled transcriptome data were converted to gene symbols. Survival time and status, age, gender, clinical stage of GC, and other clinical information were extracted and documented. We compiled a list of aberrantly expressed lncRNAs from the GC transcriptomes downloaded from Ensembl (http://asia.ensembl.org). M^6^A-related lncRNAs associated with GC were identified using co-expression network analysis in R software, and genes with a correlation coefficient > 0.4 and a *P*-value < 0.001 were selected. Interactions between m^6^A modifications and GC-associated lncRNAs were visualized. M^6^A-related lncRNAs differentially expressed between GC patients and healthy controls were screened with limma in R and genes at a false discovery rate (FDR) < 0.05 and log2 fold change (FC) filter =1 were included.

### Construction of m^6^A-related lncRNA pairs in GC

Differentially expressed m6A-related lncRNAs were paired by expression pattern ranks. Each lncRNA’s expression level was converted to its rank within the same sample. Expressions of paired lncRNAs were ranked using a 0-or-1 matrix equation: C = lncRNA-A + lncRNA-B. C is defined as 1 if the expression level of lncRNA A was higher than that of lncRNA B, otherwise C is defined as 0. After two cycles of expression ranking, lncRNA pairs with a stable expression order, whether C = 0 or 1, in 20-80% of all patient samples, were selected as stable m^6^A-related lncRNA pairs.

### Identification of m^6^A-related prognostic lncRNA pairs and construction of a prognostic risk model

We integrated m^6^A-related lncRNA pairs with overall survival data from the TCGA cohort with limma. The combined gene expression and survival data were subjected to univariate Cox regression analysis to identify prognostic lncRNA pairs, and those with a *P* < 0.001 were regarded as gene candidates. These genes were tested in the 1000-times-repeated LASSO-Cox regression model, of which those with a frequency of over 100 times were included for Cox proportional hazard regression analysis to construct a multigene signature. ROC curves analysis was performed to obtain the highest (area under the curve) AUC value, which determined the optimal risk model for prediction. The average risk score of each patient was calculated using the optimal model (the highest AUC) established, and the best predictive cutoff for risk stratification was the point at which the sum of sensitivity and specificity was maximal in ROC curves. Patients were assigned to low- and high-risk groups according to the optimal risk score cutoff, and 1-, 2-, and 3-year AUC values of the risk model were calculated to validated prediction accuracy.

### Validation of the prognostic risk model

We performed the Kaplan-Meier survival analysis to validate the accuracy of this prognostic model in discriminating the survival difference between low- and high-risk groups. Cox regression analysis for potential associations between clinicopathological features and the riskScore (the coefficient multiplied by the expression of each lncRNA pair) was carried out to evaluate the independence of the model. The *Chi*-square test was used to compare the AUCs for the difference in survival prediction between the model versus other clinicopathological biomarkers, and results were visualized in heatmaps. The Wilcoxon symbolic rank-sum test was used to examine the association between the riskScore and clinicopathological features.

### Tumor mutational burden (TMB) and somatic mutation analyses

We assessed tumor mutations in the high- and low-risk groups with ggpubr in R. Kaplan-Meier survival analysis was performed for survival differences between high- and low-TMB GC specimens stratified by a surv-cutpoint determined by the suivminer package in R. Somatic mutations in the high- versus low-risk groups were explored with maftools in R and visualized on oncoplots.

### Comparison of tumor-infiltrating immune cell subpopulations between risk groups

We employed TIMER, XCELL, QUANTISEQ, MCPCOUNTER, EPIC, CIBERSORT-ABS, CIBERSORT algorithms for immune infiltration estimations to assess differences in immune cell subpopulations between low- and high-risk patients. The difference in immune cell landscape between the two risk groups was assessed using the Wilcoxon symbolic rank-sum test.

### Relationship between biomarkers for ICIs and the lncRNA pairs in the risk model

We explored the relationship between the lncRNA pairs used in the prognostic model and the expression levels of known biomarkers for ICI treatment monitoring with ggpubr in R. We also assessed the performance of this model in predicting the efficacy of single or combined use of PD1 and CTLA4 inhibitors in high- and low-risk groups, and the results were summarized in violin plots. The analyses were based on the cased in the TCGA cohort with clinical information as tissue ID, age, sex, grade, stage, and follow-up time, etc. Other information as clinical treatments (chemotherapy and/or immunotherapy, before or after surgery) were unavailable.

### Statistical analysis

R software version 4.0.2 was utilized for all statistical analyses. Kaplan-Meier survival curves were plotted with the log-rank test. We employed the *Chi*-square test to compare differences in clinicopathological characteristics between the low- and high-risk groups. The Spearman rank correlation test was performed for correlation analysis. The significance level was set at *P* < 0.05.

## Results

### Identification of differentially expressed m^6^A-related lncRNAs in GC

**Fig. 1:**
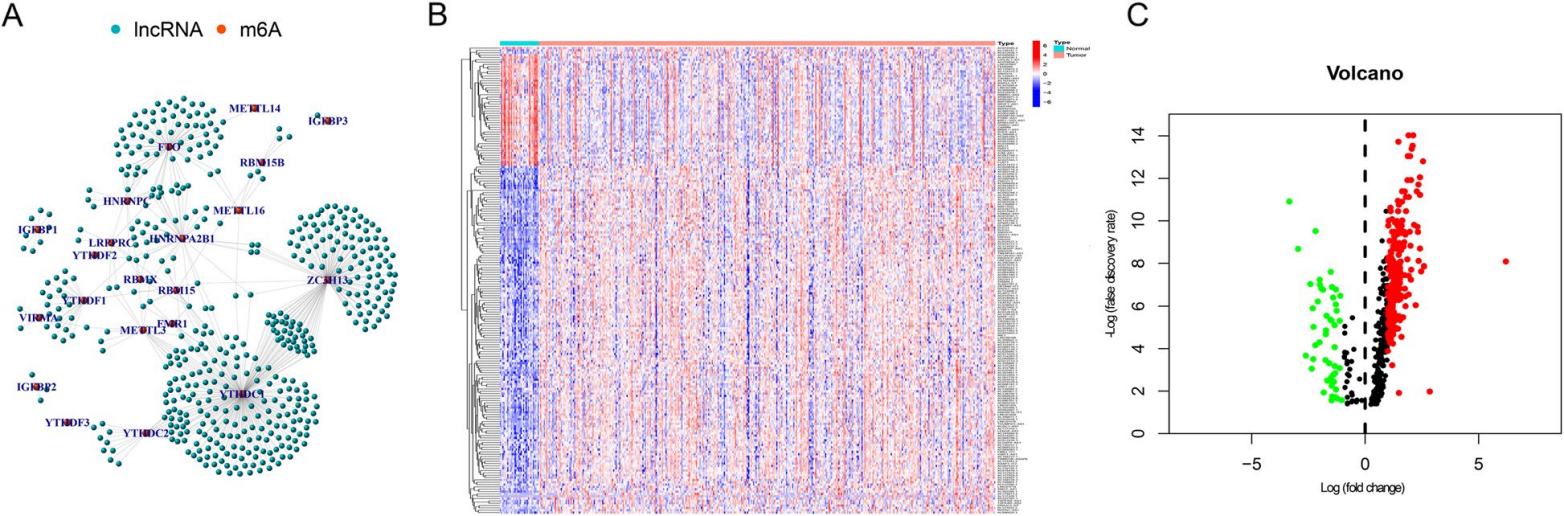
Identification of m^6^A-related lncRNAs between GC and healthy samples. **A** Co-expression network analysis shows interactions between m6A modifications (red node) and differential lncRNAs (blue node). **B** The volcano plot and **C** the heatmap demonstrate upregulated and downregulated m^6^A-related lncRNAs in GC

Initially, 489 m^6^A-related lncRNAs were identified using co-expression network analysis, and a complete network of relationships between m^6^A modifications and GC-associated lncRNAs was plotted (Fig. 1A). Next, 288 m^6^A-related lncRNAs were found differentially expressed in GC, including 49 downregulated genes and 239 upregulated ones (Fig. 1B and C, and Supplementary Additional file 1).

### Construction of the risk model based on m^6^A-related prognostic lncRNA pairs

**Fig. 2:**
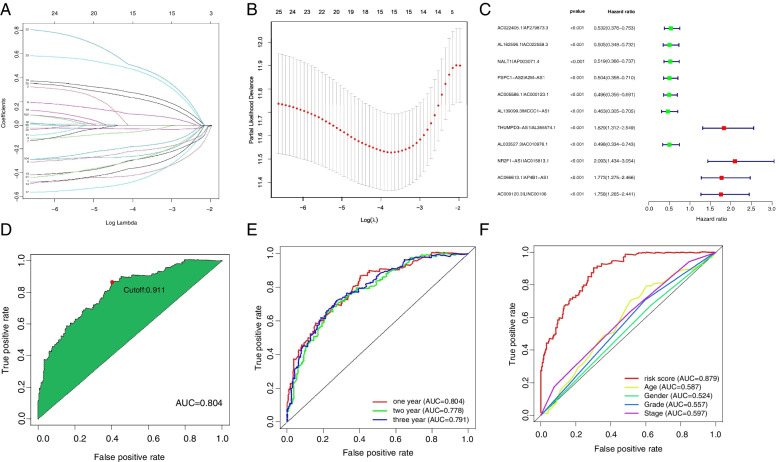
Construction of a risk model based on m^6^A-related prognostic lncRNA pairs. **A**, **B** LASSO regression analysis reveals 20,602 m^6^A-related prognostic lncRNA pairs associated with GC, 11 of which have prognostic value. **C** A risk model is constructed based on the 11 prognostic gene pairs using multivariate Cox regression. **D** The optimal cutoff calculated for risk stratification is 0.911. **E** A time-dependent ROC curve analysis shows an excellent accuracy of the risk model in 1-, 2-, and 3-year survival predictions. **F** The risk model shows greater accuracy in 1-, 2-, and 3-year survival predictions than other clinical indicators

Based on 288 m^6^A-related lncRNAs, 20,602 m^6^A-related differential lncRNA pairs were primarily identified, and 11 pairs associated with patient survival were identified using the LASSO algorithm (Fig. 2A and B) and incorporated into a Cox proportional hazard model to construct a prognostic gene signature (Fig. 2C). The ROC curve analysis showed that the AUC of the new model for risk stratification was 0.804, with the best cutoff of 0.911 (Fig. 2D). Patients were divided into low- and high-risk groups according to the best cutoff. The 1-, 2-, and 3-year AUCs for overall survival prediction in GC were 0.804, 0.778, and 0.791 (Fig. 2E), compared to the AUCs for the performances of age, gender, cancer grading, and clinical staging in survival prediction of 0.587, 0.524, 0.557, and 0.597, respectively, suggesting a superior prediction of this model (Fig. 2F).

### The risk model serves as an independent prognostic indicator for GC

**Fig. 3:**
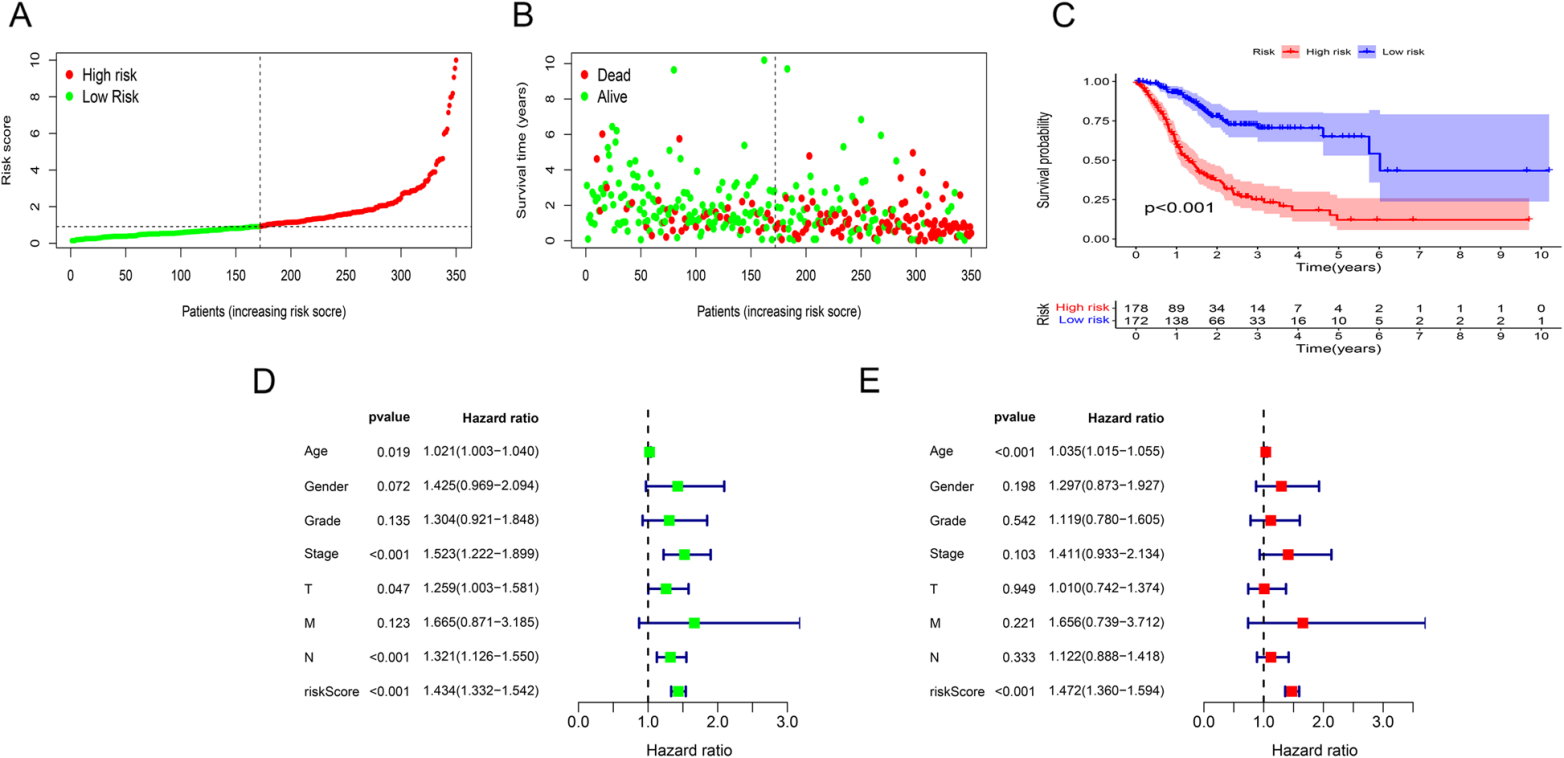
The median riskScores and survival outcome of the low- and high-risk groups. **A** The median riskScores of low- and high-risk groups. **B** The survival outcome of low- and high-risk groups. **C** High-risk GC patients reveal worse survival compared to low-risk patients. **D** Univariate Cox regression shows that the risk model has a significant association with overall survival. **E** Multivariate Cox regression demonstrates the risk model as an independent prognostic indicator of poor outcome in GC

In the TCGA cohort, 438 GC cases who had complete prognostic information were included in our analysis (Supplementary Additional file 2). All tissue samples were collected during surgical resections or biopsy procedures. Except for 27 cases with missing information, we had 56, 130, 181, and 44 patients with stage I-IV GC, respectively, according to the 7th edition of the AJCC. The median follow-up was 595 days (range, 7-2197 days). Based on the best cutoff, 178 patients were classified into the high-risk group and 172 into the low-risk group. The average riskScore and survival outcome of each group were summarized in Fig. 3A and B. The Kaplan-Meier survival analysis showed that the overall survival of low-risk patients was significantly extended versus high-risk patients (*P* < 0.001) (Fig. 3C). Univariate and multivariate Cox regression of risk ratio revealed that the riskScore could act as an independent risk indicator for overall survival prediction (univariate Cox regression: HR 1.434, 95%CI 1.332-1.542, *P* < 0.001; multivariate Cox regression: HR1.472, 95%CI 1.360-1.594, *P* < 0.001) (Fig. 3D and E).

### TMB and somatic mutation analyses in the high- versus low-risk groups

**Fig. 4:**
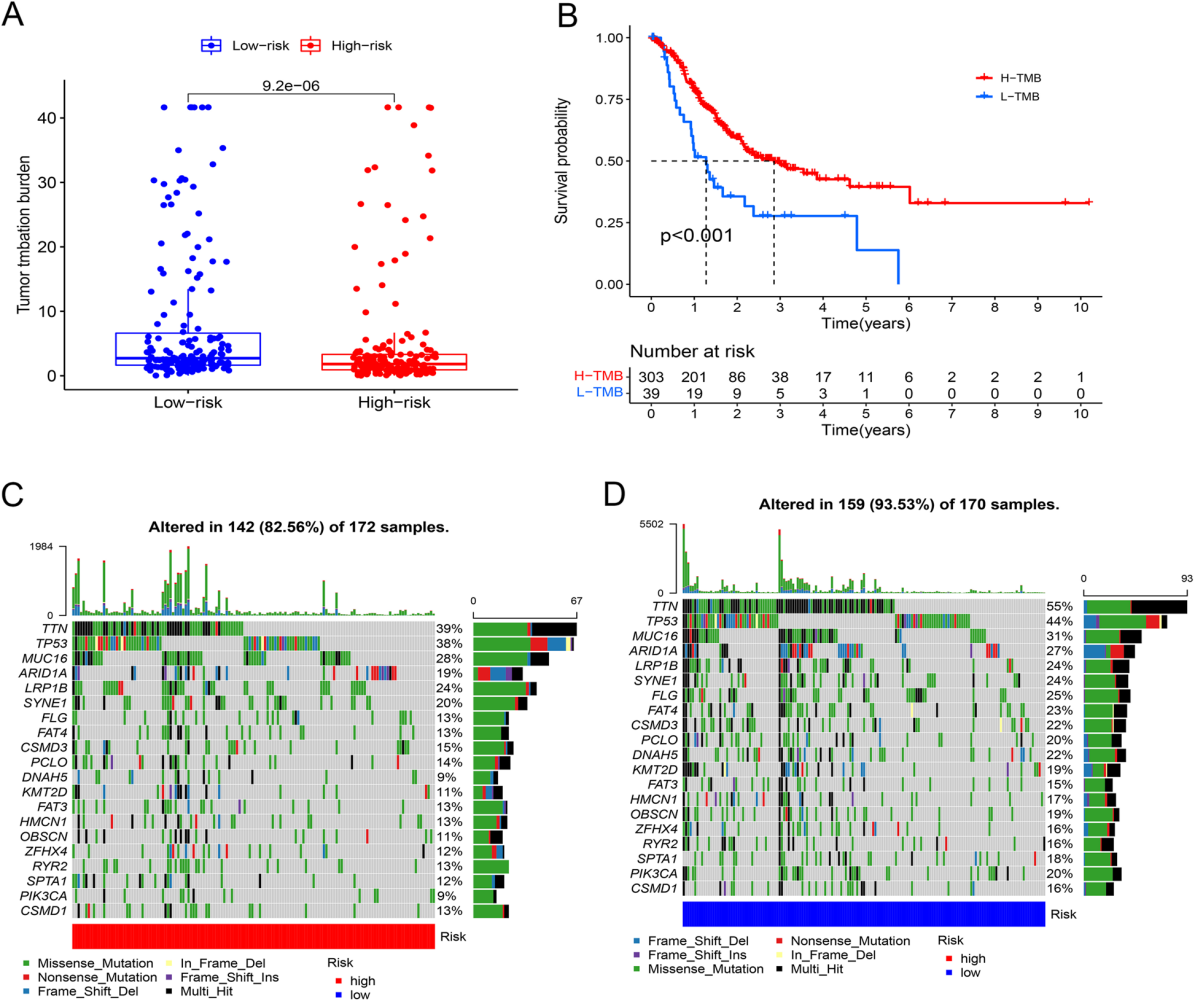
TMB and somatic mutations between the low- and high-risk groups. **A** The TMB analysis reveals high TMB in the low-risk group versus the high-risk group. **B** The Kaplan-Meier survival analysis shows longer overall survival of high-TMB patients compared to low-TMB patients. **C** Somatic mutations in the high versus low-risk groups. **D** List of somatic mutations in the low-risk group

The TMB analysis showed more frequent mutations in the low-risk group versus the high-risk group (*P* = 0.0095) (Fig. 4A). Better survival was observed in high-TMB patients (*n* = 303) versus low-TMB patients (*n* = 39), classified upon their TMB estimates, which was consistent with better overall survival in low-risk patients (Fig. 4B). In somatic mutation analysis, a higher somatic mutation rate of 91.01% was found in the low risk group compared to 84.31% in the high risk group, which were most common in *TTN* (51% vs. 42%), *TP53* (42% vs. 39%), and *MUC1*6 genes (34% vs. 24%) (Fig. 4C and D).

### The M^6^A-related lncRNAs-based risk model predicts immune cell landscape associated with GC risk

**Fig. 5:**
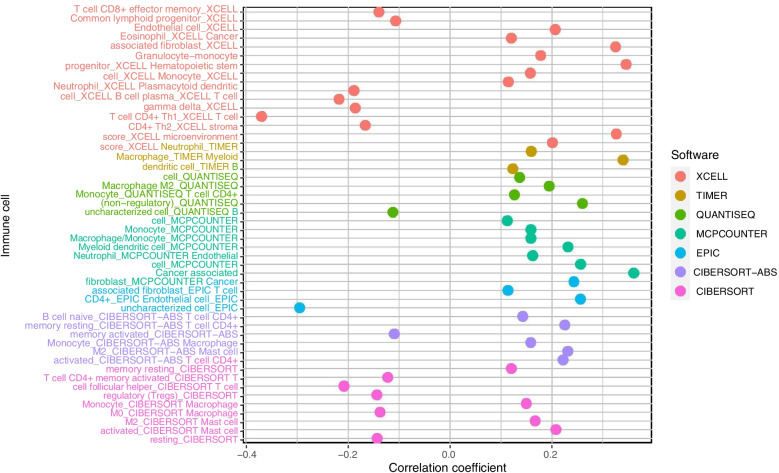
Alterations in the immune cell infiltration discriminated by the M^6^A-related lncRNA-based risk model are associated with GC risk

**Fig. 6:**
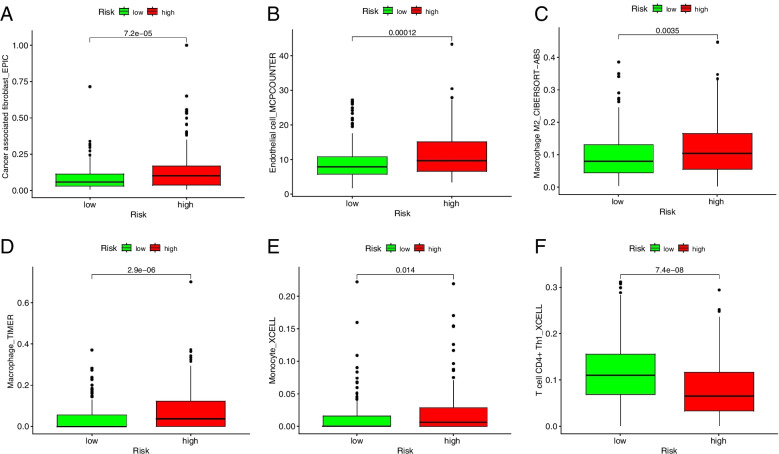
Infiltration of tumor-infiltrating immune cells in the low- versus high-risk groups. **A** to **E** Increased infiltration of cancer-associated fibroblasts, endothelial cells, macrophages, M2 macrophages, and monocytes in the high-risk group. **F** High CD4^+^ Th1 cell infiltration, for anti-tumor immune response, in the low-risk group

We assessed the potential relationships of immune cell subpopulations with GC risk to explore whether the m^6^A-related prognostic lncRNA pairs used in the risk model were involved in activities in the tumor immune microenvironment (TIME). A significant correlation was observed between alterations in the immune cell landscape and increased GC risk (Fig. 5). The differential analysis revealed markedly increased infiltration of cancer-associated fibroblasts, endothelial cells, macrophages, particularly M2 macrophages, and monocytes in high-risk patients, and high CD4^+^ Th1 cell infiltration, for anti-tumor immune response, in low-risk patients (Fig. 6A to F).

### Differences in immune-related genes between low- versus high-risk GC patients

**Fig. 7:**
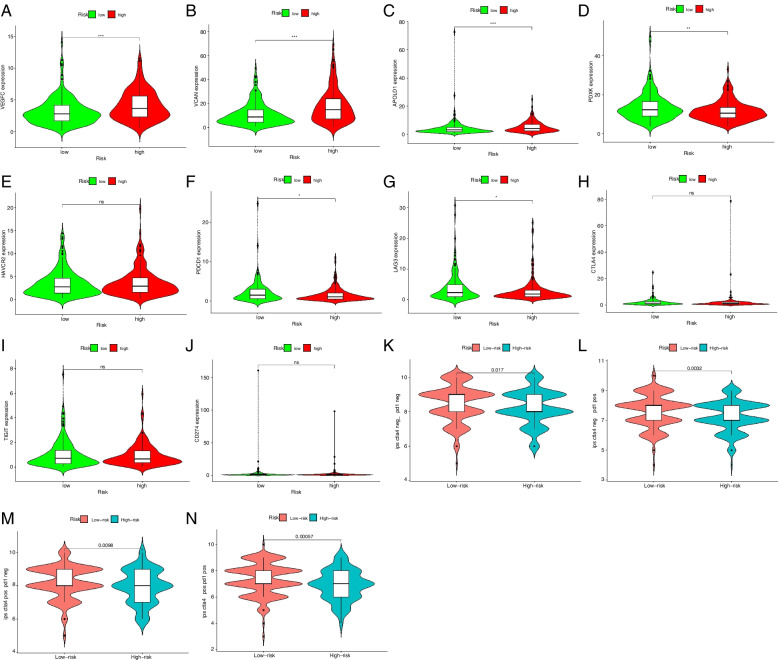
Differences in immune-related and immune checkpoint genes in low- versus high-risk GC patients. **A** to **C** *VEGFC*, *VCAN*, and *APOLD1* gene expressions are upregulated in the high-risk group. **D** *PDXK*, **F** *PD-1*, and **G** *LAG3* was highly expressed in the low-risk group, whereas **E** *HAVCR2(TIM3)*, **H** *CTLA4*, **I** *TIGIT*, and **J** *PD-L1* show no significant differences in expression levels between low- versus high-risk groups. **K** The efficacy of the common ICI treatments, *CTLA4* and *PD1* inhibitors, in the high- and low-risk groups using immunoscores. Low-risk patients receiving **L** single *PD-1* inhibitors or **M** *CTLA4* inhibitors or **N** their combination have better responses than high-risk patients. *P < 0.05, **P < 0.01, ***P < 0.001

As immunotherapy is the common treatment for GC, whether the m^6^A-related lncRNAs used in the model were associated with biomarkers for ICI treatment monitoring was examined. We found higher expressions of immune-related genes as *VEGFC*, *VCAN*, and *APOLD1* in the high-risk group and increased *PDXY* expression in low-risk patients (Fig. 7A to D). We also analyzed the differential expressions of frequently detected immune checkpoint genes as *PD-1(PDCD1)*, *PD-L1(CD274)*, *CTLA4*, *TIM3(HAVCR2)*, *LAG3,* and *TIGIT* between the two risk groups, of which *PD-1* and *LAG3* expressions were significantly upregulated in the low- versus high-risk groups (Fig. 7E to J). The analysis of immunotherapy efficacy showed that either single use of anti-PD1 or CTLA therapy or their combination had better efficacy in low-risk patients than those achieved in high-risk patients (Fig. 7K to N).

## Discussion

Although the roles of m^6^A lncRNA modification in facilitating tumor occurrence and development have been reported in several studies, the current knowledge concerning its role in GC development and progression remains limited. Alterations in malignant behaviors of tumor cells by m^6^A regulators have been reported to be an important mechanism responsible for tumor progression [22]. However, whether the mechanism to maintain tumor cell growth and survival is lncRNA-dependent remains unknown because of limited data on m^6^A lncRNA modification in particular cancer types, including GC. In the current study, a prognostic risk model was constructed based on m^6^A-related lncRNA pairs selected using the LASSO algorithm. The risk scoring model (the median riskScore) for risk stratification was confirmed based on ROC curves. We found m^6^A-related prognostic lncRNA pairs used in the risk model were associated with not only patient survival but immune cell infiltration and alterations in immune-related genes, contributing to a better knowledge of lncRNA biomarkers for GC prognosis and immunotherapies.

In this work, we integrated transcriptome and clinical data of GC patients from TCGA, identified m^6^A-related prognostic lncRNAs associated with GC prognosis, and constructed lncRNA pairs using a 0-or-1 matrix. Finally, a risk model for GC risk was developed based on these prognostic lncRNA pairs using a 1000-times-repeated LASSO regression model proposed by Sveen et al. [23]. Prognostic gene pairs were selected upon their frequency rather than intersections in the 1000 times of random stimulation for a more accurate prediction. Here are the improvements in modeling we made: the best cutoff for risk prediction and an optimal risk model selected using the highest AUC for more accurate prognostic prediction. All these added to the credibility of our results. Our risk model has been shown to effectively discriminate high-risk patients who may develop GC, as well as specific clinicopathological features and tumor-infiltrating immune cell landscape. Besides, we utilized a 0-or-1 matrix upon expression order instead of expression level to identify differentially expressed lncRNAs more efficiently, either upregulated or downregulated. Moreover, high TMB has been shown to have associations with good prognosis and enable GC efficacy prediction, thus can be used as a biomarker [24]. The somatic mutation analysis highlighted the great majority of mutations in *TTN* and *MUC16* genes in both the high- and low-risk groups, which have been proven to show significant associations with GC prognosis and offer TMB prediction or efficacy prediction in immunotherapy [25]. In this study, we also found higher TMB in the low-risk group, suggesting a relationship of high TMB with favorable survival.

In the TIME, m^6^A modification may change the patterns of tumor-infiltrating immune cells being recruited remotely, suppressing response to immunotherapy and ultimately driving tumor cell proliferation and survival and tumor progression in patients. The effect of m^6^A modification on immune cells in the TIME is critical for immune escape and patient outcome [26]. The efficacy of immunotherapy, particularly PD-1/PD-L1 inhibitors, in various cancers has been widely explored and discussed. A robust anti-cancer immune response can be restored via blocking immune checkpoint receptors and their ligands, thus increasing immune-mediated tumor clearance in the TIME [19, 26, 27]. For far too long, little has been written on m^6^A modification and tumor-infiltrating immune cell landscape in cancers, even though it is known that the efficacy of immunotherapy and patient outcome are closely related to immune cell infiltration [28]. The latest study ascertained that reduced m^6^A modification by knocking out methyltransferase genes, *Mettl3* and *Mettl14*, enhanced response to PD-1 inhibitors in murine colorectal carcinoma cell line CT26 [29]. This finding provides a clue that an m^6^A-related signature may be important to shape the immune cell landscape in high-risk populations and predict response to immunotherapy, which are crucial for enhancing the success rate of immunotherapy. We compared the difference in immune cell subpopulations between low- and high-risk patients stratified by the risk score cutoff and assessed the association of m^6^A-related lncRNA pairs selected for the risk model with immune cell infiltration using TIMER [30], XCELL [31], QUANTISEQ [32], MCPCOUNTER, EPIC, CIBERSORT [33], CIBERSORT-ABS algorithms [34]. These gene pairs showed tight associations with high CD4^+^ T cell, macrophage, monocyte, and myeloid dendritic cell infiltration. Cancer-associated fibroblasts, endothelial cells, hematopoietic cells, resting memory CD4^+^ T cells in patients at high risk of GC were significantly abundant versus low-risk cases. There were more macrophages, macrophage-monocyte lineage cells, monocytes, neutrophils, and CD4^+^ Th1 cells infiltrated in tumor tissues of low-risk patients. These findings suggest that m^6^A lncRNA modification may be involved in the inhibition of the anti-cancer immune response in high-risk patients. Our findings were in line with the studies of m^6^A-related lncRNA signature in bladder cancer [35], which based on 9 lncRNAs. Compared with that study, our risk model was constructed based on 11 lncRNA pairs, which may reduce the batch corrections and increase the accuracy of the analyses.

Recent reports have shown that only a small group of cancer patients can benefit from ICIs [36], which calls for new biomarkers for ICI response prediction or even related adverse event prediction. In this study, we identified three immune-related genes, *VEGFC*, *VCAN*, and *TNFSF*, overexpressed in tumor tissues of high-risk patients and *PDXY* and *APOLD1* upregulated in the low-risk group. This model is apt at discriminating aberrant immune checkpoint genes in different risk groups. Among the immune checkpoint genes, *PD-1*, *PD-L1*, *CTLA4*, *TIM3*, *LAG3*, and *TIGIT* frequently detected in most ICI research [36, 37], we found *PD-1* and *LAG3* expressions were upregulated in the high-risk group versus the low-risk group. We also found that either single use of anti-PD1 or CTLA therapy or their combination had better efficacy in low-risk patients than those achieved in high-risk patients. That means low-risk patients can also benefit from ICIs, whatever the specific agents or combination regimens. The findings above suggest that this risk model is an excellent option for monitoring immunotherapeutic efficacy.

## Conclusion

In summary, we developed a risk model based on m^6^A-related prognostic lncRNA pairs for GC risk using the optimal modeling algorithms. It effectively shapes the tumor-infiltrating immune cell landscape and predicts the efficacy of immunotherapy in low- and high-risk patients, which can be used as an accurate and independent predictor for GC risk. The 11-m^6^A-related lncRNA signature used in the model is worthy of further exploration to offer therapeutic targets for better immunotherapy. However, more information of the TCGA cohort, such as medication schedules, surgical records, and pathological reports, are unavailable but essential for insightful analysis, which is expected in our future publication in a timely manner. Large-sample, multicentre studies of assessment of m^6^A-related lncRNA-based risk model in GC prognosis prediction are needed.

## Supplementary Information

**Additional file 1.**

**Additional file 2.**

## Data Availability

All data generated or analyzed during this study are included in this published article [and its supplementary information files]. The raw clinical and transcriptome (or RNA-seq) data used in this study were all available from the TCGA database (https://portal.gdc.cancer.gov/repository?facetTab=files&filters=%7B%22op%22%3A%22and%22%2C%22content%22%3A%5B%7B%22op%22%3A%22in%22%2C%22content%22%3A%7B%22field%22%3A%22cases.primary_site%22%2C%22value%22%3A%5B%22stomach%22%5D%7D%7D%2C%7B%22op%22%3A%22in%22%2C%22content%22%3A%7B%22field%22%3A%22cases.project.program.name%22%2C%22value%22%3A%5B%22TCGA%22%5D%7D%7D%2C%7B%22op%22%3A%22in%22%2C%22content%22%3A%7B%22field%22%3A%22cases.project.project_id%22%2C%22value%22%3A%5B%22TCGA-STAD%22%5D%7D%7D%2C%7B%22op%22%3A%22in%22%2C%22content%22%3A%7B%22field%22%3A%22files.analysis.workflow_type%22%2C%22value%22%3A%5B%22HTSeq%20-%20FPKM%22%5D%7D%7D%2C%7B%22op%22%3A%22in%22%2C%22content%22%3A%7B%22field%22%3A%22files.data_category%22%2C%22value%22%3A%5B%22transcriptome%20profiling%22%5D%7D%7D%2C%7B%22op%22%3A%22in%22%2C%22content%22%3A%7B%22field%22%3A%22files.data_type%22%2C%22value%22%3A%5B%22Gene%20Expression%20Quantification%22%5D%7D%7D%5D%7D).

## Abbreviations

AUC: Area under the curve
GC: Gastric cancer
lncRNAs: Long noncoding RNAs
m6A: N6-methyladenosine
TCGA: The Cancer Genome Atlas
TIME: Tumor immune microenvironment
VIRMA: Vir-like m6A methyltransferase associated
ALKBH5: Alkylation repair homolog protein 5
